# A Decad(e) of Reasons to Contribute to a PLOS Community-Run Journal

**DOI:** 10.1371/journal.pgen.1005557

**Published:** 2015-10-05

**Authors:** Gregory P. Copenhaver, Gregory S. Barsh

**Affiliations:** 1 The University of North Carolina at Chapel Hill, Chapel Hill, North Carolina, United States of America; 2 Stanford University School of Medicine, Stanford, California, United States of America

## Abstract

Will be part of a Tenth Anniversary Collection

Whether it's the number of primate digits, the number of Vishnu avatars, or the number of items on David Letterman's lists for the last 30 years, humans have an almost inordinate fascination with the number ten. That fascination has been especially apparent at *PLOS* these past few months, as we and two of our sister journals celebrate our tenth anniversary in close succession. Recalling the wonderful "Ten Simple Rules" articles published in *PLOS Computational Biology*, we at *PLOS Genetics* are publishing a Tenth Anniversary Collection of our Top Ten Research Articles published over the last ten years.

Setting aside, momentarily, the silliness of decimal system obsession, it is worth some reflection and discussion about exactly what qualifies an article to be in the "Top Ten." Among all articles published by *PLOS Genetics* in the last ten years, we considered the number of citations, the number of views, the number of times an article had been shared via a social media link from our website, and the number of downloads. There are obvious and inherent biases in each of these metrics. For example, a paper that reports groundbreaking research in a specific area could be read by everyone in that community but may be at a disadvantage relative to a less influential paper read by a fraction of a much larger community. Similarly, recently published papers haven't had as much opportunity to accumulate citations, views, and/or downloads compared to older papers. These considerations notwithstanding, our Top Ten Collection encompasses a broad range of publication dates, organisms, and approaches and reflects the central mission of the journal: to publish great science across all areas of genetics that is of broad interest and is by working scientists, for working scientists.

The last half of that mission is why we care so deeply about *PLOS Genetics*. There are other broad-interest journals that publish great science, but what makes *PLOS Genetics* special is that the group of people who make the editorial decisions are from the same groups that actually do the work. As *PLOS* changes the publishing landscape and launches initiatives that aim to advance science across disciplines, the strength of *PLOS Genetics* and the other community-run *PLOS* journals will rest with the investment made by the scientists who take on the role of editors.

With that perspective, and with a nod to Mr. Letterman's unique brand of satire, here are our top ten reasons to contribute to a *PLOS* community-run journal. Proverbial drum roll, please…

10 We are free to read and use—*and everybody loves free stuff*.
9 Unlike other for-profit greed-heads, we’re not for profit, *so you can feel virtuous*.
8 We’re a community journal—*kind of like a modern day hippie commune*, *dig it*?
7 All of our editors actually still do science—*unlike some other journals…just saying*.
6 We make the website awesome—*because we know scientists are dazzled by shiny stuff*.
5 Our staff rock—*seriously*, *visiting the offices is like attending a hipster convention*.
4 Being an editor at *PLOS Genetics* is kind of like being on a grant review panel—*except it's easier and you actually get to make decisions*.
3 Like Craig Venter's genome, our standards for research and publishing are developed from the bottom up—*but with fewer errors*.
2 We’re always looking for ways to help you reach your readership—*anyone up for* PLOS *aerial drones*?
1 Because it’s YOUR journal.

## Editorial Commentaries on the *PLOS Genetics* Tenth Anniversary Collection Articles

### Population Structure and Eigenanalysis

Nianjun Liu, David B. Allison

University of Alabama, United States of America

Detecting and quantifying population structure (including admixture) is important in a variety of situations, such as medical genetics, subspecies classification, forensic studies, genetic barrier detection, and evolutionary research. However, this is challenging even with genetic data. Perhaps the first important question is whether population structure is implied by the data under study. There are methods available for such purpose. However, they generally do not provide rigorous statistical tests based on analytic derivations. Principal components analysis (PCA) has been widely used in genetic study for almost 40 years. It has been one important tool for exploring population structure, especially in medical genetics, in which genetic association studies are usually performed. However, most work on PCA in genetic studies is applied instead of methodological. For example, there is not a formal way to select the number of principal components used in the study. In the paper “Population Structure and Eigenanalysis,” Patterson, Price, and Reich addressed these questions by combining PCA with modern statistical theory [1]. In addition to the formal test for population structure and additional structure, the method can estimate the data size needed to detect structure. The method is based on a solid statistical foundation. This gives researchers justification and confidence to use the tests and/or PCA in their studies.

### A Mutation in the Myostatin Gene Increases Muscle Mass and Enhances Racing Performance in Heterozygote Dogs

Joseph S. Takahashi

University of Texas Southwestern Medical Center, Howard Hughes Medical Institute, United States of America

Everyone loves dogs, and the genetics of dogs is especially appealing, as Jasper Rine and Elaine Ostrander have argued, because of the selective breeding of dogs by humans to produce breeds that are morphologically and behaviorally diverse in the extreme [2]. This paper showed that a loss-of-function mutation in the *myostatin* gene in whippets increases muscle mass, and importantly, heterozygous carriers of this mutation are associated with faster speed in competitive racing. This was the first paper to link a *myostatin* mutation with athletic performance—thus, its significance.

### Genome-Wide Association Scan Shows Genetic Variants in the *FTO* Gene Are Associated with Obesity-Related Traits

Gregory S. Barsh

Stanford University School of Medicine, United States of America

To many human geneticists and epidemiologists, the search for genomic variation that underlies quantitative traits is something of a holy grail, providing insight into diseases that impact public health, realizing the promise and power of genetic epidemiology, and fulfilling a nearly century-old prediction of Sir Ronald Fisher. The manuscript from Abecasis and colleagues is one of three high-profile papers published in 2007 that exemplify that search for one of the most quantitative of traits: adiposity [3]. This work was especially notable because of its precision and rigor: using an isolated population (from an ancient small town in Sardinia) reduced the effect of potential confounding variables, and the results were compelling.

### Genetic Variation and Population Structure in Native Americans

Chris Tyler-Smith

The Wellcome Trust Sanger Institute, United Kingdom

In 2007, when this paper was published, genome-wide studies of human genetic diversity were just beginning. High-density single nucleotide polymorphism (SNP) chips were not widely available, so this and other early studies used a few hundred short tandem repeats (STRs, also known as microsatellites). Previously, only five Native American populations had been examined in this genome-wide way, so this paper, adding 25 more, was the first to provide a wide genomic overview of Native Americans, ranging from the Chipewyan in Canada to the Huilliche in Chile [4]. It confirmed the low genetic diversity in the Americas and allowed structure within these continents to be examined. The chief conclusions favored a single major colonization via the Bering Strait and hinted at the use of coastal routes within the Americas but found only limited correlations with language. Subsequent studies have of course updated the technology and added further levels of complexity, including inferring additional prehistoric colonizations limited to the northern areas that were not covered here and enriching our understanding with ancient DNA. But overall, our view remains that defined in 2007. What more could we ask of a paper?

### Population Genomics of Parallel Adaptation in Threespine Stickleback Using Sequenced RAD Tags

Gregory S. Barsh

Stanford University School of Medicine, United States of America

In "Wonderful Life," Stephen Jay Gould suggested that if the "tape of life" were rewound and replayed, things might turn out very differently. Gould's argument was based on fossils preserved in the Burgess Shale about 508 million years ago and, like many evolutionary theories, difficult to test: there is only one Burgess Shale. The same is not true, however, for the threespine stickleback, which has become one of the evolutionary biologist’s favorite "model organisms," precisely because there are multiple populations that have adapted and evolved independently from oceanic ancestors to thrive in freshwater lakes. The manuscript from Cresko and colleagues applies genomic technology to sticklebacks from five populations in Alaska, two oceanic and three freshwater, to explore independent "tapes of life" over thousands of years, and comes to a remarkable conclusion [5]. The freshwater populations show many regions across the genome that have evolved independently and in parallel, in some cases exhibiting signatures of divergent selection (in which the same regions in freshwater populations show large differences from oceanic populations) and in some cases exhibiting balancing selection (in which the same regions in freshwater populations maintain unexpectedly high levels of heterozygosity). The appeal of this work is both in its theory—who doesn't like to think about evolution—and in its application, since the authors show how next-generation sequencing technology can be applied to any natural population to study similar questions. A wonderful life, indeed.

### Genomewide Analysis of PRC1 and PRC2 Occupancy Identifies Two Classes of Bivalent Domains

Bas van Steensel

The Netherlands Cancer Institute, The Netherlands

Polycomb group (PcG) complexes are repressive complexes with key gene-regulatory functions during development. By genome-wide mapping of these complexes in mouse and human embryonic stem (ES) cells, the authors extended an earlier observation that PcG complexes bind to unmethylated CpG islands [6]. The authors identified sequence features (in addition to CG-richness) that predict localization of PcG complexes to a remarkable degree. Furthermore, they discovered that so-called bivalent chromatin domains come in two “flavors” that differ in the presence of PcG subcomplexes. This study has helped to lay the foundation of our current understanding of the genomic targeting of PcG complexes in mammals. It is no surprise that the remarkable connection of PcG complexes to the widely studied (but poorly understood) CpG islands has sparked a booming field of research.

### A Flexible and Accurate Genotype Imputation Method for the Next Generation of Genome-Wide Association Studies

Nicholas J. Schork

J. Craig Venter Institute, United States of America

Contemporary human genetics research has been greatly enhanced not only by technical advances in, e.g., high-throughput genotyping and sequencing technologies but also by clever algorithms that exploit the data generated by these technologies. Nowhere is that clearer than in the methods developed to facilitate the thousands of genome-wide association studies (GWAS) pursued in the last decade or so. GWAS have generally been expensive to pursue, so strategies that extend the number of genetic variants that can be interrogated in an efficient and cost-effective manner have had a significant impact on their execution. The paper by Howie, Donnelly, and Marchini describes a method for “imputing” or assigning genotypes to individuals at loci that have not been directly assayed by available genotyping chips, essentially extending the number of variants capable of being tested for association in GWAS without having to resort to expensive and time-consuming laboratory assays [7]. The authors do this by exploiting linkage disequilibrium patterns between alleles at loci not interrogated by a GWAS genotyping chip and alleles at loci directly assayed on the chip using a sophisticated and comprehensive strategy. This strategy has three important features: a method for accommodating the potentially diverse genetic backgrounds of individuals in a GWAS, a method for making genotype assignments more reliable than previous imputation methods, and a very efficient algorithm for carrying out the relevant computational operations. The embodiment of this strategy was made available to the research community by the authors in the often-used IMPUTE2 computer program. The availability of IMPUTE2 has enabled hundreds of GWAS to be more comprehensive than they otherwise would have been and, importantly, has also motivated additional research into genotype imputation as well as related phenomena such as haplotyping and the influence of admixture and genetic background on association studies.

### Exploring Microbial Diversity and Taxonomy Using SSU rRNA Hypervariable Tag Sequencing

Jonathan A. Eisen

University of California Davis, United States of America

Today, one of the hottest topics in all of biology is "The Microbiome"—or the communities of microbes that live in and on various plants and animals (e.g., the human microbiome) and also that occupy particular niches (e.g., the home microbiome). The growing appreciation of such microbial communities can be traced to a few factors, but perhaps the most important one is the continuing exponential developments in DNA sequencing. This is because DNA sequencing is a critical gateway into characterizing complex communities of microbes. However, DNA sequencing developments alone did not revolutionize microbiome studies: perhaps even more important were developments in laboratory and computational tools that allowed one to leverage DNA sequencing to deal with the massive scale of microbial diversity (e.g., thousands of species per small sample). This paper by Huse et al. represents a key development in this field because they showed how by integrating computational and laboratory tools one could rapidly and cheaply characterize microbial communities using "short read" sequencing methods [8].

### A Molecular Phylogeny of Living Primates

Anne D. Yoder

Duke University, United States of America

Published in 2011, "A Molecular Phylogeny of Living Primates" by Perelman et al. has now been cited more than 300 times—a proud accomplishment for the authors, and for *PLOS Genetics*. Much of the paper's appeal lies with the fact that we humans are primates, and thus, it focuses on our phylogenetic home address [9]. But this is hardly the first (or most recent) phylogeny of primates. It is true that the majority of articles citing this paper concentrate on primate-specific issues, but other organismal studies, ranging in focus from rodents, to whales, to bats, and even dinosaurs, have also taken notice. The paper has also been referenced in more general papers on genomic architecture, evolutionary rates, and phylogenetic reconstruction. So what is the magic? My thinking is that it appeared at a special moment in the science of phylogeny reconstruction, when it was becoming relatively easy to sequence lots of genes for lots of species and, very importantly, to analyze these large datasets with new efficiency. Given that genomic and computational tools have become even more efficient, powerful, and economical, it will be fascinating to see the next "definitive" primate phylogeny and in what details, if any, it will differ from Perelman et al.

### Organised Genome Dynamics in the *Escherichia coli* Species Results in Highly Diverse Adaptive Paths

Josep Casadesús

Department of Genetics, University of Seville, Spain

Juliet's question "What's in a name?" may be appropriate when asked of many taxonomic categories, and some answers can be awkward. An example is the notion of bacterial species. Under the name *Escherichia coli*, for instance, we group a range of bacterial variants with disparate lifestyles, from the friendly commensals of our intestine to deadly pathogens. By comparing the genomes of 20 natural isolates of *E*. *coli* and one isolate of the related species *Escherichia fergusonii*, Touchon et al. [10] provided a meaningful answer to the question "What's in the name *E*. *coli*?" The average *E*. *coli* genome has some 4,700 genes, and a core genome of some 2,100 genes is found in all strains. At the core genome, isolates that may have diverged 25–30 million years ago remain 98% identical at the DNA sequence level as a consequence of gene conversion, which may be 100 or more times higher than the mutation rate. While the size and gene content of the core genome remains more or less constant, the overall genome size can differ up to 30% from one isolate to another, reflecting the occurrence of high rates of gene acquisition and loss. Hence, the existence of a core genome justifies the existence of the taxonomic category *E*. *coli*. Nevertheless, no strain can be considered representative of the species. Gene scramble at the peripheral genome is a ceaseless source of evolutionary innovation, including the formation of novel pathogenic variants. The stark clarity of the study, its significance in bacterial evolution, and its relevance for human health may explain the high impact of this paper on the scientific community.
